# Efficacy of combined surgery and pembrolizumab for the treatment of pulmonary large cell carcinoma: a case report

**DOI:** 10.3389/fimmu.2024.1500996

**Published:** 2024-12-23

**Authors:** Linguo Gu, Hongzuo Chen, Zhenkun Xia, Bei Qing, Yunchang Yuan

**Affiliations:** Department of Thoracic Surgery, Second Xiangya Hospital, Central South University, Changsha, China

**Keywords:** pulmonary large cell carcinoma, surgery, PD-L1, pembrolizumab, case report

## Abstract

Pulmonary large cell carcinoma (LCC) is a rare and aggressive subtype of non-small cell lung cancer (NSCLC) with poor prognosis. Surgical resection remains the cornerstone of treatment for resectable LCC; however, its efficacy is limited in advanced stages, necessitating adjuvant therapies to reduce postoperative recurrence risk. Recent advances in immunotherapy have shown promising survival benefits. Here, we present a case of LCC successfully treated with a combination of surgery and pembrolizumab. A 56-year-old male smoker, diagnosed with LCC and staged as T2N1M0 postoperatively, developed recurrent disease one month after surgery, as evidenced by enlarged left hilar and mediastinal lymph nodes on chest CT. The patient received adjuvant chemotherapy and immunotherapy, guided by high PD-L1 expression. However, after three cycles, chemotherapy was discontinued due to severe side effects, and pembrolizumab monotherapy was initiated. After 21 cycles, there was substantial regression of the mediastinal and hilar lymph nodes. The patient remained progression-free after 24 cycles of treatment. This case underscores the potential of combining surgical resection with immunotherapy as an effective strategy not only for resectable LCC but also for other rare NSCLC subtypes with high PD-L1 expression.

## Introduction

Pulmonary large cell carcinoma (LCC), a rare and distinct subtype of non-small cell lung cancer (NSCLC), constitutes approximately 10% of all NSCLC cases (1, 2). In clinical practice, LCC is distinguished by its highly aggressive behavior and association with poor prognosis. Across all stages of LCC, the 5-year overall survival rate and 5-year disease-free survival rate are approximately 35% and 27%, respectively, with a significant proportion of relapses occurring within the first two years of follow-up (3). Traditional surgical resection, as the cornerstone of treatment for LCC patients, offers potential benefits for a subset of individuals (4). However, in patients with advanced disease, the therapeutic efficacy of surgery is constrained, and the incidence of postoperative recurrence remains alarmingly high. Additionally, adjuvant chemotherapy, modeled on regimens established for small cell lung cancer (SCLC), has shown potential in improving patient outcomes and enhancing overall survival rates (5). However, the potential benefit of adjuvant chemotherapy for LCC patients remains contentious, and to date, there is no unified expert consensus to guide clinical treatment.

Recent studies have shown that neoadjuvant combination immunotherapy has the potential to markedly enhance the major pathological response (MPR) and pathological complete response (pCR) rates in patients with NSCLC, while maintaining a favorable toxicity profile (6). Although LCC is a subtype of NSCLC, there is currently a lack of conclusive evidence to support the applicability of immunotherapy for patients with LCC. The potential benefits of immunotherapy for patients with LCC remain largely unexplored, and consequently, it is seldom considered in clinical practice. Only a handful of case reports have explored the application of immunotherapy in LCC patients (7). Here, we present a case of LCC that was successfully treated with a combination of surgical resection and pembrolizumab, a programmed cell death-1 (PD-1) immune checkpoint inhibitor.

## Case report

**Figure 1:**
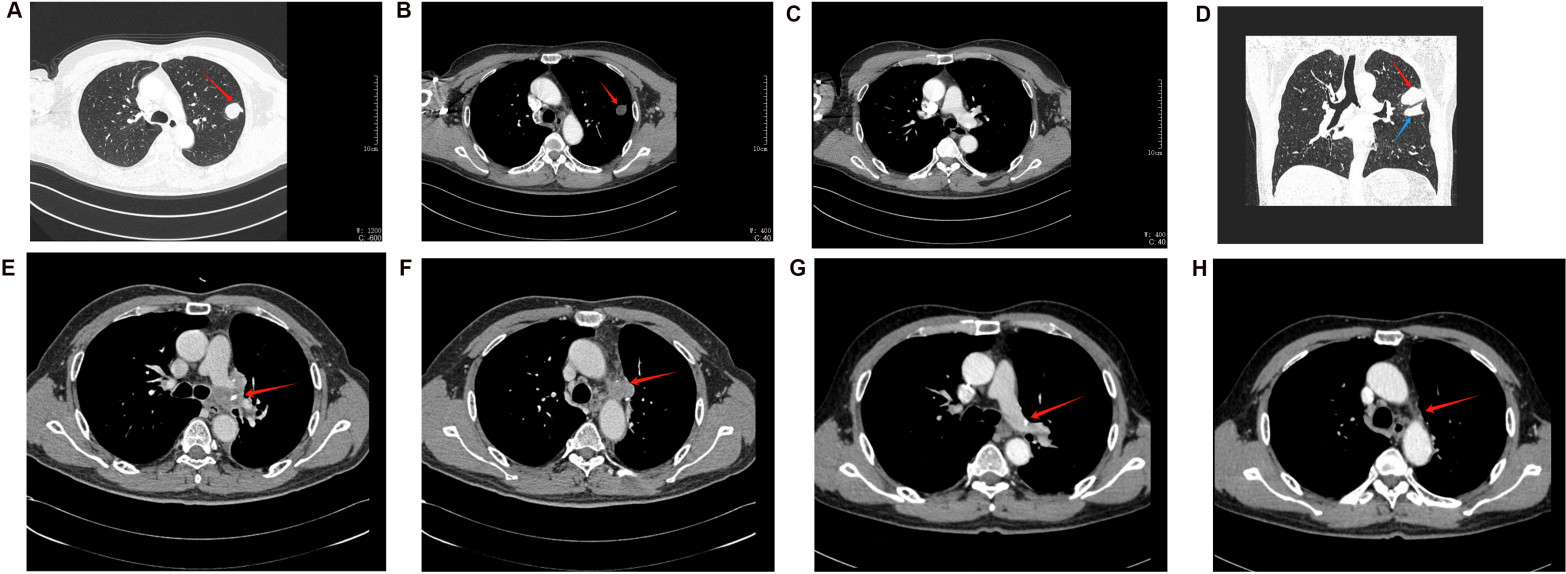
The dynamic changes in CT imaging. **(A–C)** CT results of the patient’s initial examination. **(A)** The lung window reveals a round-like mass in the left upper lung, characterized by an irregular shape but relatively well-defined borders (indicated by the red arrow). **(B)** The mediastinal window image of the same plane reveals uneven density (red arrow). **(C)** The mediastinal window of a different plane shows no significant enlargement of the mediastinal lymph nodes. **(D)** The CT coronal image taken 2 months later shows an increase in the size of the primary lesion (red arrow), along with the appearance of a new small lesion (blue arrow). **(E, F)** Follow-up CT one month after the patient’s surgery. **(E)** displays an enlarged, irregular lymph node in the left hilum (red arrow). **(F)** shows similarly irregularly enlarged lymph nodes in other regions of the mediastinum (red arrows). **(G, H)** CT follow-up images of the patient after receiving pembrolizumab treatment. Compared to panel **E,** panel **G** shows a reduction in the size of the hilar lymph nodes, with signs of regressive changes. Similarly, compared to panel **F**, panel **H** shows a reduction in the size of the mediastinal lymph nodes, indicating the effectiveness of pembrolizumab treatment.

**Figure 2:**
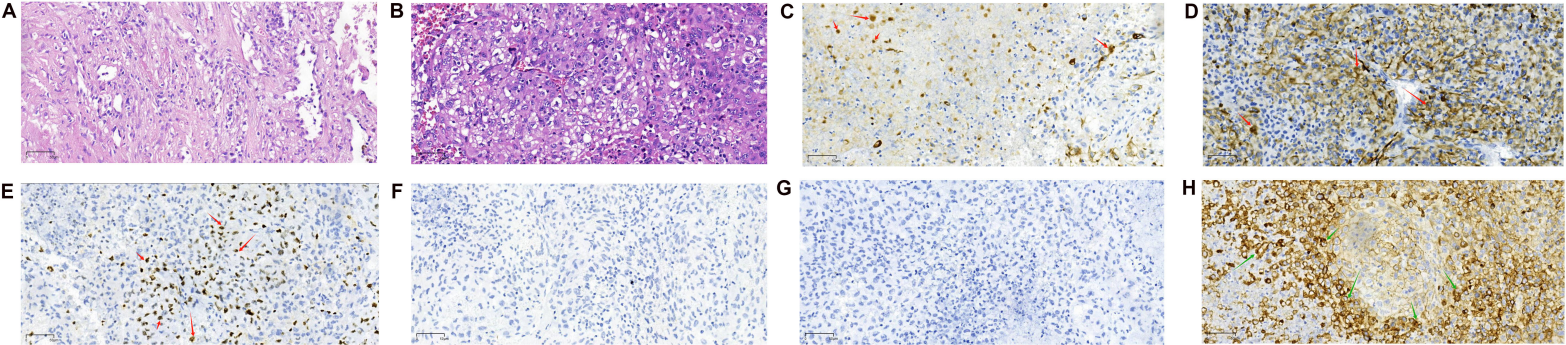
Pathological images (magnification: 20X objective lens). **(A)** Hematoxylin and eosin (H&E) staining of the biopsy specimen obtained via CT-guided aspiration, showing necrotic tissue and fibrous proliferation. **(B)** H&E staining of the surgically resected mass. The tumor cells are large, irregular in shape, with prominent nuclear pleomorphism and abundant cytoplasm, which may exhibit vacuolar changes. The nuclei are large, with coarse and uneven chromatin, and prominent nucleoli. The tumor shows a disorganized arrangement of cells, lacking distinct glandular or squamous structures. **(C–G)** Immunohistochemical Staining Results. **(C)** Immunohistochemical staining for Cytokeratin (CK, Abcam, ab7753) is positive. **(D)** Immunohistochemical staining for Cytokeratin 7 (CK7, Abcam, ab181598) is positive. CK and CK7 positivity suggests that the tumor is of epithelial origin, with a high possibility of non-small cell lung cancer. **(E)** The Ki-67 (Antigen Ki-67, Abcam, ab15580) immunohistochemical staining shows positive results. **(F)** P40 (Abcam, ab137691) immunohistochemical staining was negative. **(G)** TTF-1 (Thyroid Transcription Factor-1, CST, 23192) immunohistochemical staining was negative. The positivity of Ki-67, along with the negativity of P40 and TTF-1, suggests that the tumor is unlikely to be squamous cell carcinoma (due to P40 negativity) or adenocarcinoma (due to TTF-1 negativity). However, the high proliferative activity indicated by Ki-67 positivity implies that this tumor may be a subtype of NSCLC with high malignancy potential. This immunohistochemical profile is commonly observed in LCC, which typically presents as an undifferentiated tumor with a lack of distinct differentiation features. Therefore, based on these markers, the diagnosis of LCC is further supported. **(H)** PD-L1 immunohistochemical staining demonstrated high PD-L1 expression, with a tumor proportion score exceeding 60%.

A 56-year-old male smoker was admitted to our hospital with a persistent cough and hemoptysis lasting for over a month. Chest computed tomography (CT) revealed a quasi-circular mass in the left upper lobe of the lung (**Figures 1A, B**), however, there was no significant enlargement of the mediastinal lymph nodes (**Figure 1C**). A CT-guided percutaneous biopsy of the lung mass was performed, and hematoxylin and eosin (HE) staining revealed necrotic tissue and fibrous hyperplasia, but a definitive diagnosis could not be established (**Figure 2A**). Two months later, the patient was readmitted for surgical intervention due to the rapid progression of the primary mass following the initial fine-needle aspiration biopsy. A subsequent CT scan revealed significant growth of the primary lesion, accompanied by the emergence of a new, smaller lesion in the adjacent area (**Figure 1D**). Following a comprehensive preoperative evaluation, the patient underwent a left upper lobectomy combined with systematic lymph node dissection. Postoperative histopathological analysis of both nodules confirmed stage IIIA LCC (T2N1M0) (**Figure 2B**). Molecular testing did not identify any actionable gene mutations. Immunohistochemical analysis revealed positive staining for cytokeratin (CK) and CK7 expression, with a Ki-67 index of 30%. Both P40 and thyroid transcription factor-1 (TTF-1) were negative (**Figures 2C–G**). Notably, PD-L1 immunohistochemistry demonstrated high expression, with a tumor proportion score (TPS) exceeding 60% (**Figure 2H**). The patient recovered uneventfully after surgery with no complications. However, a follow-up chest CT one month later revealed enlargement of the left hilar and mediastinal lymph nodes, indicating tumor recurrence (**Figures 1E, F**). The patient subsequently received three cycles of adjuvant chemotherapy combined with pembrolizumab. Owing to intolerance to chemotherapy-induced side effects, the patient continued with pembrolizumab monotherapy for 21 additional cycles. This regimen led to a rapid regression of the mediastinal and hilar lymph nodes. CT scans showed significant reduction in lymph node size, and a complete response was observed (**Figures 1G, H**). The patient remained progression-free throughout 24 cycles of treatment, and pembrolizumab was well-tolerated with no severe adverse events or signs of disease progression.

## Discussion

LCC is an exceptionally rare primary lung tumor and a subtype of NSCLC. Characterized by neuroendocrine features, LCC exhibits a higher degree of malignancy compared to typical NSCLC (8). Even after surgical resection, patients remain at a significant risk of recurrence (9). Additionally, LCC is prone to developing resistance to chemotherapy, further complicating treatment and posing substantial challenges in management (10). In the early stages of NSCLC, curative-intent surgery remains the cornerstone of treatment and offers the best chance for a cure. Lobectomy or pneumonectomy accompanied by systematic nodal dissection is typically preferred, as it may extend overall survival (11). However, previous studies have shown that even among stage I patients, the 5-year survival rate following surgical resection varies significantly, ranging from 18% to 88% (12). The efficacy of platinum-based conventional chemotherapy regimens varies considerably across different studies, with response rates ranging from 45% to 70% (13).

Pembrolizumab is a specific monoclonal antibody that inhibits programmed cell death protein 1 (PD-1). PD-1, upon binding to its ligands, programmed death-ligand 1 (PD-L1) and programmed death-ligand 2 (PD-L2), which are present on tumor cells or within the tumor microenvironment, typically transmits inhibitory signals that suppress T cell function, thereby facilitating tumor evasion of immune surveillance (14). As an anti-PD-1 monoclonal antibody, pembrolizumab blocks the interaction between PD-1 and its ligands, PD-L1/PD-L2, thereby restoring T cell immune activity and enhancing the body’s immune response against tumor cells (15). The results from the KEYNOTE-024 and KEYNOTE-042 trials have demonstrated the superior efficacy of pembrolizumab compared to conventional platinum-based chemotherapy as a first-line treatment for advanced non-small cell lung cancer (NSCLC) with a PD-L1 tumor proportion score (TPS) of 50% or higher, in the absence of EGFR/ALK mutations (16, 17). Although the potent efficacy of pembrolizumab in NSCLC has been well established, limited research has focused on its applicability to LCC. Key questions remain unanswered, including the optimal timing for initiating treatment, the appropriate duration of therapy, and whether pembrolizumab should be combined with chemotherapy or other treatment modalities.

As an immunotherapeutic agent, pembrolizumab is associated with certain adverse effects, which are inevitable to some extent. These primarily include anemia, neutropenia, diarrhea, nausea and vomiting, fatigue, alopecia, and anorexia (18–20). Although the patient reported in our case has not experienced adverse effects to date, it is inevitable that some side effects may emerge during subsequent follow-up. The integration of diverse treatment modalities has significantly advanced the management of lung cancer (21). Due to the encouraging outcomes observed in stage IV disease, immunotherapy is increasingly being explored in earlier stages as consolidation therapy alongside surgical intervention (22). In the case of this patient, a remarkable therapeutic response was achieved after 24 cycles of pembrolizumab treatment. Although theoretical studies suggest that large cell carcinoma may respond to immunotherapy, there are currently no authoritative clinical guidelines recommending the use of pembrolizumab for this indication (23). Our case report illustrates a successful treatment with the immunosuppressant Pembrolizumab for a patient with post-operative recurrence of large cell carcinoma. This finding underscores the limitations of surgical treatment alone, as patients may still experience recurrence, suggesting that immunotherapy could be a valuable consideration in such scenarios. Furthermore, this successful treatment case lays the foundation for future use of immunotherapy in the management of LCC, as well-conducted and scientifically rigorous randomized controlled trials are essential to validate this approach.

The patient was successfully managed with a combination of surgical resection and pembrolizumab. However, in the absence of randomized controlled trials specifically targeting LCC, the optimal therapeutic strategy for this malignancy remains poorly defined. This case highlights the potential of integrating surgical intervention with immunotherapy as a promising treatment approach, not only for resectable large cell lung cancer but also for other rare non-small cell lung cancer subtypes exhibiting high PD-L1 expression.

## Data Availability

The original contributions presented in the study are included in the article/supplementary material. Further inquiries can be directed to the corresponding authors.

## References

[1] Pelosi G Barbareschi M Cavazza A Graziano P Rossi G Papotti M. Large cell carcinoma of the lung: a tumor in search of an author. A clinically oriented critical reappraisal. Lung Cancer. (2015) 87:226–31. doi: 10.1016/j.lungcan.2015.01.008 25620799

[2] Driver BR Portier BP Mody DR Deavers M Bernicker EH Kim MP. Next-generation sequencing of a cohort of pulmonary large cell carcinomas reclassified by world health organization 2015 criteria. Arch Pathol Lab Med. (2016) 140:312–7. doi: 10.5858/arpa.2015-0361-OA 26430808

[3] Iyoda A Hiroshima K Moriya Y Iwadate Y Takiguchi Y Uno T. Postoperative recurrence and the role of adjuvant chemotherapy in patients with pulmonary large-cell neuroendocrine carcinoma. J Thorac Cardiovasc Surg. (2009) 138:446–53. doi: 10.1016/j.jtcvs.2008.12.037 19619794

[4] Kinslow CJ May MS Saqi A Shu CA Chaudhary KR Wang TJC. Large-cell neuroendocrine carcinoma of the lung: A population-based study. Clin Lung Cancer. (2020) 21:e99–e113. doi: 10.1016/j.cllc.2019.07.011 31601526

[5] Wang Y Chen Y Yang Z Qian F Hu M Lu J. Different characteristics and survival between surgically resected pure and combined pulmonary large cell neuroendocrine carcinoma. Ann Surg Oncol. (2022) 29:5666–78. doi: 10.1245/s10434-022-11610-4 35543906

[6] Saw SPL Ong B-H Chua KLM Takano A Tan DSW. Revisiting neoadjuvant therapy in non-small-cell lung cancer. Lancet Oncol. (2021) 22:e501–16. doi: 10.1016/S1470-2045(21)00383-1 34735819

[7] Luo Z Zhang H Xiao Y Wang R Zhang L Huang C. Durable response to immunotherapy with antiangiogenic drug in large-cell lung carcinoma with multiple fulminant postoperative metastases: A case report. Front Oncol. (2021) 11:633446. doi: 10.3389/fonc.2021.633446 34094914 PMC8173040

[8] Kupeli M Koseoglu RD. Large cell carcinoma with adenocarcinoma in lung. J Coll Physicians Surg Pak. (2018) 28:240–2. doi: 10.29271/jcpsp.2018.03.240 29544586

[9] Pelosi G Fabbri A Cossa M Sonzogni A Valeri B Righi L. What clinicians are asking pathologists when dealing with lung neuroendocrine neoplasms? Semin Diagn Pathol. (2015) 32:469–79. doi: 10.1053/j.semdp.2015.10.009 26561395

[10] Welter S Aigner C Roesel C. The role of surgery in high grade neuroendocrine tumours of the lung. J Thorac Dis. (2017) 9:S1474–s1483. doi: 10.21037/jtd.2017.01.60 29201450 PMC5690951

[11] Zacharias J Nicholson AG Ladas GP Goldstraw P. Large cell neuroendocrine carcinoma and large cell carcinomas with neuroendocrine morphology of the lung: prognosis after complete resection and systematic nodal dissection. Ann Thorac Surg. (2003) 75:348–52. doi: 10.1016/S0003-4975(02)04118-8 12607637

[12] Chen Y Zhang J Huang C Tian Z Zhou X Guo C. Survival outcomes of surgery in patients with pulmonary large-cell neuroendocrine carcinoma: a retrospective single-institution analysis and literature review. Orphanet J Rare Dis. (2021) 16. doi: 10.1186/s13023-021-01730-7 PMC7881654 33579331

[13] Yang L Fan Y Lu H. Pulmonary large cell neuroendocrine carcinoma. Pathol Oncol Res. (2022) 28. doi: 10.3389/pore.2022.1610730 PMC9592721 36304941

[14] Herbst RS Baas P Kim DW Felip E Pérez-Gracia JL Han JY. Pembrolizumab versus docetaxel for previously treated, PD-L1-positive, advanced non-small-cell lung cancer (KEYNOTE-010): a randomised controlled trial. Lancet. (2016) 387:1540–50. doi: 10.1016/S0140-6736(15)01281-7 26712084

[15] Herbst RS Garon EB Kim DW Cho BC Gervais R Perez-Gracia JL. Five year survival update from KEYNOTE-010: pembrolizumab versus docetaxel for previously treated, programmed death-ligand 1-positive advanced NSCLC. J Thorac Oncol. (2021) 16:1718–32.doi: 10.1016/j.jtho.2021.05.001 34048946

[16] Reck M Rodríguez-Abreu D Robinson AG Hui R Csőszi T Fülöp A. Updated analysis of KEYNOTE-024: pembrolizumab versus platinum-based chemotherapy for advanced non-small-cell lung cancer with PD-L1 tumor proportion score of 50% or greater. J Clin Oncol. (2019) 37:537–46. doi: 10.1200/JCO.18.00149 30620668

[17] Mok TSK Wu YL Kudaba I Kowalski DM Cho BC Turna HZ. Pembrolizumab versus chemotherapy for previously untreated, PD-L1-expressing, locally advanced or metastatic non-small-cell lung cancer (KEYNOTE-042): a randomised, open-label, controlled, phase 3 trial. Lancet. (2019) 393:1819–30. doi: 10.1016/S0140-6736(18)32409-7 30955977

[18] Burtness B Rischin D Greil R Soulières D Tahara M de Castro G Jr.. Pembrolizumab alone or with chemotherapy for recurrent/metastatic head and neck squamous cell carcinoma in KEYNOTE-048: subgroup analysis by programmed death ligand-1 combined positive score. J Clin Oncol. (2022) 40:2321–32. doi: 10.1200/JCO.21.02198 PMC9287281 35333599

[19] de Castro G Jr. Kudaba I Wu YL Lopes G Kowalski DM Turna HZ. Five-year outcomes with pembrolizumab versus chemotherapy as first-line therapy in patients with non-small-cell lung cancer and programmed death ligand-1 tumor proportion score ≥ 1% in the KEYNOTE-042 study. J Clin Oncol. (2023) 41:1986–91. doi: 10.1200/JCO.21.02885 PMC10082298 36306479

[20] Harrington KJ Burtness B Greil R Soulières D Tahara M de Castro G Jr.. Pembrolizumab with or without chemotherapy in recurrent or metastatic head and neck squamous cell carcinoma: updated results of the phase III KEYNOTE-048 study. J Clin Oncol. (2023) 41:790–802. doi: 10.1200/JCO.21.02508 36219809 PMC9902012

[21] Hirsch FR Scagliotti GV Mulshine JL Kwon R Curran WJ Jr. Wu YL. Lung cancer: current therapies and new targeted treatments. Lancet. (2017) 389:299–311. doi: 10.1016/S0140-6736(16)30958-8 27574741

[22] Heigener DF Reck M. Immune checkpoint inhibition in non-metastatic non-small cell lung cancer: chance for cure? Drugs. (2019) 79:1937–45. doi: 10.1007/s40265-019-01222-w 31749060

[23] Lindsay CR Shaw EC Moore DA Rassl D Jamal-Hanjani M Steele N. Large cell neuroendocrine lung carcinoma: consensus statement from The British Thoracic Oncology Group and the Association of Pulmonary Pathologists. Br J Cancer. (2021) 125:1210–6. doi: 10.1038/s41416-021-01407-9 PMC8548341 34489586

